# Survival of Hypercapnic Patients with COPD and Obesity Hypoventilation Syndrome Treated with High Intensity Non Invasive Ventilation in the Daily Routine Care

**DOI:** 10.2174/1874306401711010031

**Published:** 2017-06-30

**Authors:** Thomas Blankenburg, Christin Benthin, Stefanie Pohl, Anett Bramer, Frank Kalbitz, Christine Lautenschläger, Wolfgang Schütte

**Affiliations:** 1 2^nd^ Medical Dept., Hospital Martha-Maria Halle-Doelau, D-06120 Halle, Germany; 2 1^st^ Medical Dept., Hospital Martha-Maria Halle-Doelau, D-06120 Halle, Germany; 3 Institute of Medical Epidemiology, Biostatistics, and Informatics, Martin-Luther-University Halle-Wittenberg, D-06112 Halle, Germany

**Keywords:** Chronic Obstructive Pulmonary Disease, Non-invasive ventilation, Routine care, Hypercapnia, Ventilatory insufficiency, Prognosis, Home ventilation, Routine care

## Abstract

**Background:**

Home ventilation is an effective treatment option for obesity hypoventilation syndrome (OHS). This therapy is still controversial for stable chronic obstructive pulmonary disease (COPD). A recent study showed reduced mortality for COPD patients receiving home ventilation with high inflation pressures and back-up respiratory rates [so called High Intensity non-invasive ventilation (NIV)].

**Objective:**

The purpose of this study is whether High Intensity NIV applied in the routine care of COPD and OHS patients can lead to CO_2_ reduction and survival data comparable to data from controlled studies.

**Method:**

In this prospective non interventional study fifty-one patients with COPD (FEV1 0.95l, corr. 32.8%) and 34 patients with OHS (VC 1.74l, corr. 50.7%) with chronic hypercapnic respiratory failure, who were treated with NIV were followed up for four years.

**Results:**

Elevated CO_2_ values before NIV in COPD patients (8.6kPa), and in OHS patients (8.3kPa), could be lowered significantly to the upper normal range (COPD: 5.9kPa; OHS: 5.85kPa). The one-, two-, and three-year survival rates for COPD patients were 83%, 73%, and 55%, respectively. The one-, two-, and three-year survival rates for OHS patients were 85%, 72%, and 68%, respectively.

**Conclusion:**

High intensity NIV within routine care is effective in reducing blood CO_2_ levels in COPD- and in OHS- related chronic respiratory insufficiency. The survival rates obtained here are comparable to data from controlled clinical trials on COPD.

## INTRODUCTION

Chronic respiratory failure, in severe COPD, leads to a deterioration of the quality of life and a significant worsening of prognosis [1]. While the value of non-invasive ventilation, in cases of acute COPD exacerbation, has long been undisputed [2] , home ventilation for chronic respiratory failure, caused by COPD, is not yet established. This is due to controversy in the literature. Compared with other diseases, such as obesity hypoventilation syndrome (OHS), the clinical and prognostic effects of non-invasive ventilation (NIV), in chronic respiratory failure due to COPD, are not always demonstrable [3, 4]. In studies showing benefits to prognosis and the quality of life, or a reduced hospitalisation rate in COPD, the patients were predominantly severely hypercapnic and the treatment was carried out with high pressures and high ventilation rates (so called “High Intensity NIV”) [5-10]. In studies showing no benefit of home ventilation therapy in stable COPD, the patients were either less hypercapnic, the ventilation pressures lower, or they were acute ventilatory decompensated patients who were switched to home ventilation [11-13] after acute care ventilation. Continuing artificial ventilation therapy as home ventilation, even when carried out at high pressures and high frequencies, has not shown any prognostic effect in acute respiratory insufficient COPD patients [13] . A recent study by Koehnlein *et al.* 2014 [5] showed that “High Intensity NIV” had a significant influence on mortality in a controlled trial in severely hypercapnic, stable COPD patients. Fourteen days of intense patient contact were a special feature of this study’s design. On the other hand, in chronic respiratory/ventilatory failure due to OHS, the prognostic value of non-invasive ventilation is undisputed [14-16] . In contrast to COPD, this disease is not characterised by frequent exacerbations. In an earlier study by Janssens *et al.* in 2003 [4] it was shown, for example, that in OHS, in contrast to COPD, home ventilation led to a significant reduction of hypercapnia and improved survival.

The purpose of this study is whether High Intensity NIV applied in the routine care of COPD and OHS patients can lead to CO_2_ reduction and survival data comparable to data from controlled studies. The second question is whether high intensity NIV in COPD is comparable with OHS with respect to the reduction of hypercapnia and survival.

## PATIENTS AND METHODS

### Study Design in- and Exclusion Criteria

This was a prospective non interventional single centre study performed on a centre for home ventilation in a hospital with approximately 500 beds. The recruitment period was from January to December 2011. The median follow up period was four years. The study has been approved by the local ethics committee prior to the start of the study.

We recruited all inpatients, with stable COPD and OHS, who were adjusted to NIV due to chronic respiratory insufficiency on a specialized normal ward. Patients were admitted because of daytime hypercapnia. The recruitment took place before discharge with intermittent non-invasive home ventilation. COPD was diagnosed according to the international guidelines (GOLD), based on anamnestic information, the clinical examination, and pulmonary function (post-bronchodilator- FEV_1_/FVC < 70%). OHS was diagnosed according to the current common definitions, based on a BMI > 30kg/m^2^, detection of chronic daytime hypercapnia (pCO_2_ > 6.7kPa, pH > 7.35), and characteristic symptoms [14, 15, 17]. Patients with anamnestic, clinical or Para clinical indications of exacerbation, or respiratory acidosis, were excluded. Likewise, patients with indications of decompensated left- or right-ventricular heart failure, or other conditions that could lead to chronic respiratory failure, were not enrolled in this study (Table **1**).

Arterial blood gas analysis (ABG), before NIV, was carried out in the morning, at rest, in spontaneously breathing, seated patients using blood from the hyperaemic earlobe. ABG prior to discharge was carried out, two hours after NIV, in spontaneously breathing patients. Obese patients with a relative FEV_1_ of less than 0.7 were not classified as OHS patients. Six-minute walk tests were carried out using Guyatt’s method.

### Schedule of Therapy Initiation

After appropriate instruction patients were gradually familiarised (twice daily) with the home ventilation parameters, as well as, the duration of the ventilation intervals. Only conventional mouth-nose masks and a tubing system with valve control were used. The ventilation device used was a VSIII, (Saime SA, France) and the mainly used ventilation mode applied was APCV (assisted pressure controlled ventilation). When APCV was not achievable, pressure support ventilation was accepted if normocapnia could be documented. The inspiratory pressure was chosen such that the patient did not experience any “air hunger” during inspiration and a tidal volume of, at least, 800ml was reached. The goal was a minimum Pinsp >/= 20 cm H_2_O. PEEP was applied in all OHS patients and in COPD patients with indications of obstructed exhalation (due to instability of the upper airways). In each case, the lowest possible PEEP was used. The algorithm for selecting the optimum PEEP worked by escalating/increasing PEEP until unhindered, even exhalation, and comfortable inhalation triggering for the patient, became possible. The ventilation rate was set at two breaths/min above the spontaneous respiratory rate, of the patient, for 80% of breaths. The duration of ventilation was gradually extended to at least 12h/d. The objective was to reach normocapnia during spontaneous breathing, as well as, acceptance of the therapy. If hypoxaemia was still detectable during effective ventilation, oxygen supplementation was provided in accordance with the recommendations for LTOT (GOLD). Patients were discharged when normocapnia was achieved within the period of spontaneous breathing, or when the pCO_2_ has decreased by at least 1kPa, and a steady state of spontaneous breathing pCO_2_ values was reached. A further requirement for discharge with NIV was the ability to safely handle the ventilation device, as well as, the willingness of the patient to apply the therapy to the prescribed extent. Patients who rejected this form of therapy during the titration phase, or who could not use it independently were not included in this study.

### Follow-up Examinations

Standardised, inpatient follow-up examinations were carried out, initially, four weeks after setting up NIV, then 12 weeks after the first check-up examination, and subsequently in six-monthly intervals. If no modifications in the ventilation therapy were necessary within the first year after adjustment, check-up examinations were carried out annually. During follow-up examinations complications and ventilatory effectiveness were reviewed. This included nocturnal oximetry, ABG during NIV, while spontaneously breathing at rest and under physical challenge. For these tests, patients were hospitalised for one night. Survival data were obtained in a two-step process. In the first step, treatment data of the ventilation device were collected by the device provider. Information regarding discontinuation of treatment and survival status were recorded for all patients within the four-year study period. In cases of treatment discontinuation, the reason for discontinuation or, where applicable, the date of death was medically validated in the second step. The last control examination, within the four-year study period, was recorded as the last point of contact. The average daily device use duration was calculated from the total hours of ventilation recorded in the device memory divided by the number of usage days in the first year, or by the total usage period.

### Data Management and Statistics

Patient data and ventilation specific data were entered after enrolment into a database and continuously updated over the course of the study. The number of ventilation hours was read out and recorded from the ventilation device by the device provider after 12 months of ventilation, or after discontinuation/death of the patient. Plausibility checks were performed at an individual case level, as well as, at the database level, and if necessary, corrections were made. Implausible data that were impossible to clarify were recorded as missing. Continuous data were reported as mean and standard error as the sample data were normally distributed. Categorical data were recorded as n and %. Statistical tests were performed using t-tests for group differences. Survival was documented using the univariate Kaplan-Meier-analysis survival analysis. Statistical analyses were performed using the statistics package SPSS 17.0 (IBM, Germany).

## RESULTS

### 1 Patient Characteristics

The defined patient groups have characteristic anthropometric and pulmonary function data. Pulmonary function analysis in COPD patients showed a severe degree of obstructive respiratory disorder, as well as, severe distension of the lung. The median BMI shows that the patients were predominantly obese; therefore, this patient cohort was phenotypically characterised as predominantly bronchitic. OHS patients, with a vital capacity of 50.7%, characteristically have considerable restrictive functional impairment (Table **2**). The extent of hypercapnia (COPD 8.6kPa; OHS 8.3kPa) indicates severely hypercapnic patients with respiratory insufficiency.

### 2 Ventilation Effectiveness

Ventilation parameters are shown in Table **3** below. High inspiration and high effective pressures (Pinsp-PEEP) were achieved. Inspiration pressures and ventilation rates (VR) were similar between COPD and OHS patients (with PInsp of 22 vs. 22cm H_2_O and VR of 15.8/min *vs*. 15.3/min). Ventilation parameters differed in PEEP values (COPD: 2.3 cm H_2_O; OHS: 5.3 cm H_2_O). The low PEEP and the high proportion of patients with a PEEP of zero result from the PEEP titration scheme applied here and the control of exhalation through a valve system.

On average patients were discharged with a prescription of 12h/d of intermittent assisted ventilation. The prescribed ventilation durations did not differ significantly between COPD and OHS patients.

The effectiveness of ventilation therapy was comparable between the two groups according to the pCO_2_ values measured at discharge (Table **4**). The clearly increased values before ventilation in COPD (8.6kPa), as well as in OHS (8.3kPa), could be lowered significantly to the upper normal range (COPD: to 5.9kPa; OHS: to 5.85kPa). Normocapnia was achieved under ventilation therapy in COPD in 70.6% of cases, and in OHS in 61.8% of cases. With home ventilation COPD patients achieved a CO_2_ reduction of 1.7kPa (corresponding to 19.8% of the initial value). OHS patients achieved a CO_2_ reduction of 1.5kPa (corresponding to 18.1% of the initial value). The extent of metabolic hypercapnia compensation, as measured by HCO_3_ and BE values, could be reduced towards normal, in both groups, but not to within the normal range. The BE and bicarbonate values at discharge were 6 and 30 mmol/l, in COPD patients and 4.5 and 29.4mmol/l, in OHS patients. Overall, we found that under ventilation the ventilation specific ABG parameters (pCO_2_, BE, HCO_3_) normalised slightly more in OHS patients than in COPD patients. Prior to ventilation OHS patients had a pO_2_ of 7.55 kPa and oxygen saturation (SO_2_) of 86% more hypoxic than COPD patients who had an average pO_2_ of 8.25 kPa and SO_2_ value of 90%. The proportion of patients that had to receive long-term oxygen supplementation, in addition to ventilation, was the same in both groups. Sufficient oxygenation was achieved in both groups. The physical capacity of COPD and OHS patients, as measured by the 6-minute walking distance test, was significantly limited. During the course of inpatient treatment, the walking distance of both groups significantly increased from 193m to 271m in COPD patients, and from 192m to 238m in OHS patients. The increase in walking distance of COPD patients was significantly higher (78m) than OHS patients (42m).

### 3 Survival Analysis

**Fig. (1):**
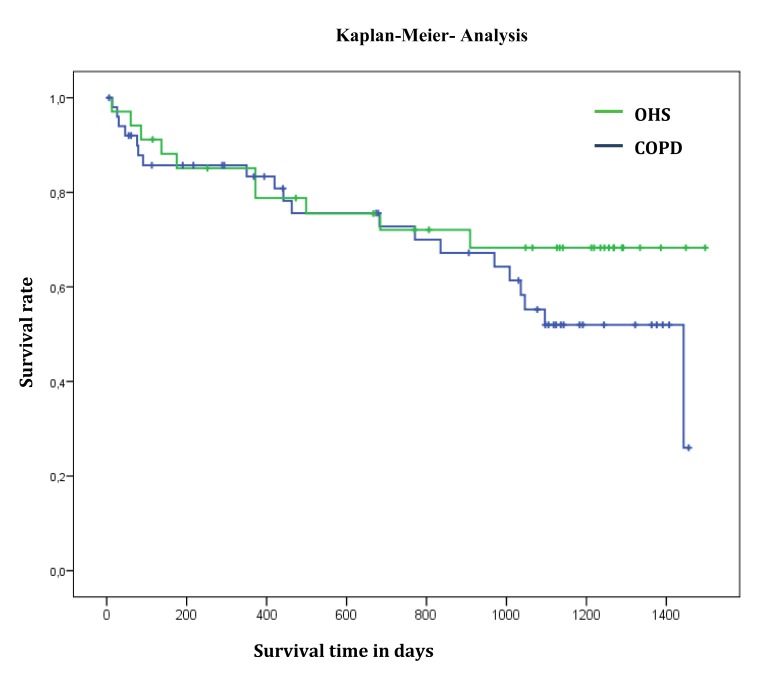
Survival of COPD and OHS patients: Univariate Analysis Kaplan-Meier.

The one-, two-, three-, and four-year survival rates (SR) for artificially ventilated COPD patients were 83%, 73%, 55%, and 26%, respectively. One- and two-year survival rates of OHS patients were identical at 85% and 72%, three- and four-year survival rate was 68%, which was better than that of COPD patients (Table **5** and Fig. **1**).

## DISCUSSION

### Discussion of the Ventilation Settings and Blood Gas Analysis

In this study, stable COPD and OHS patients were treated with home artificial ventilation at high inspiration pressures and high ventilation rates. The bicarbonate and the BE values in COPD and OHS patients (COPD 32.9mmol/l and 8; OHS 31.7mmol/l and 7.5) indicate, in addition to a normal pH value, that these patients have chronic respiratory insufficiency. The ventilator settings corresponded to ventilation parameters that were used in earlier studies of COPD and OHS patients, who used home ventilation with the aim of reducing hypercapnia due to respiratory insufficiency in chronically ill patients [5, 9, 10, 18, 19]. Patients were artificially ventilated with high effective pressures of 18cm H_2_O (COPD), and 17cm H_2_O (OHS). The difference between COPD and OHS patients was due to the need for a higher PEEP in the OHS group. PEEP was applied in all OHS patients and in COPD patients with indications of obstructed exhalation (due to instability of the upper airways). In each case, the lowest possible PEEP was used. The algorithm for selecting the optimum PEEP worked by escalating/increasing PEEP until unhindered, even exhalation, and comfortable inhalation triggering for the patient, became possible. This achieved a relatively low PEEP in OHS patients, and a PEEP of 0 cm H_2_O in 41% of COPD patients, which in our view promotes compliance.

Within the first year of this study, discontinuation rates of 18% and 21% were documented in COPD and OHS patients, respectively. These discontinuation rates are to be regarded as high; however, they were not primarily caused by a lack of acceptance of the high ventilation pressures used, but rather predominantly by a reduced disease perception and a lack of willingness to carry out an elaborate and costly therapy on their own. With this therapy, effective, short-term CO_2_ reduction was achieved in both groups. The improvement of ventilation specific parameters (pCO_2_, BE, HCO_3_) was comparable in both groups. Thereby, we can substantiate that in COPD patients with chronic respiratory failure under home ventilation, with high ventilation pressures, a recompensation of the respiratory insufficiency can be achieved that is similar to OHS patients.

### Discussion of Survival Rates

The one-, two-, and three-year SR for artificially ventilated COPD patients were 83%, 73%, and 55%, respectively. The one-year SR of COPD patients, in our study, is comparable with data from Köhnlein *et al.* 2014 [5] (one-year SR 88%) who used similar ventilation parameters, but a slightly lower intensive follow-up regimen. However, patients in our study, were older (median age 68.7 *vs*. 62.2 years) and physically more limited (6-minute walk distance: 196 *vs*. 226m). Although the patients we analysed were more severely hypercapnic compared to Köhnlein *et al*.’s [5] study (pCO_2_ 8.6kPa vs. 7.8kPa), we found a similar reduction in CO_2_ of 1.7kPa (corresponding to 19.8% of the initial value) after initiation of artificial ventilation. In a study by Borel *et al.* 2014 [10], the three-year SR for artificially ventilated COPD patients was approximately 40% (mortality rate 45.5% in 44.7 months) and, therefore, lower than in this study. The patients were approximately of the same age (68.7 vs. 68 years), but hypercapnia was lower in Borel *et al.* [10] at 48.5mm Hg (7.3kPa). However, in the aforementioned study, 50% of patients were adjusted to home ventilation after acute care ventilation. In our study, we excluded patients who received acute care ventilation. Apart from the proportion of patients, receiving acute care ventilation it was notable that the effective ventilation pressure (Pinsp – PEEP) in Borel *et al.* [10] was significantly lower at 11cm H_2_O compared to our study (19.7cm H_2_O). In a study on chronically respiratory insufficient patients, by McEvoy *et al.* 2009 [6], a lower 3-year SR (approximately 48%) under NIV can be inferred. Compared to our study the patients were of the same age; however less obese (BMI 25.4 *vs*. 32.4kg/m^2^), and had slightly lower FEV_1_ values (0.55l, corr. 23.1% *vs*. 0.95l, corr. 32,8%). The initial pCO_2_ was 6.9kPa, and, therefore, lower than in the patients presented here. It is remarkable that in this study, as well, the effective ventilation pressure was only 7.8cm H_2_O, and that the average duration of ventilation was 4.5h/d. We assume that in the studies of McEvoy *et al.* [6] and Borel *et al.* [10] less hypercapnic patients were examined, and that the ventilation intensities (with respect to the ventilation pressure and duration of ventilation) were lower than in our study. Thus, it can be concluded that in severely hypercapnic COPD patients, ventilation therapy with high pressures and rates appears to be associated with improved survival.

In the OHS patients of our study, ventilation therapy also leads to a significant reduction of pCO_2_ values from 8.3 to 5.8kPa. Therefore, we achieved greater CO_2_ reduction (-4kPa) compared to a recent study by Masa *et al.* 2015 [20] (-1kPa) that reported their CO_2_ reduction as clinically relevant. In comparison with a similar study by Ojeda *et al.* in 2015 [21], the 3-year survival rates for OHS patients presented here are approximately 15% worse. In this aforementioned study, a 3-year SR of 83% was achieved. Therapy compliance and average daily use duration were better in the Spanish study (5.7h/d *vs*. 5.3h/d) compared to our study. To what extent a longer use duration, or other disease characteristics (degree of functional impairment, age, or extent of hypercapnia) contribute to prognosis, ultimately remains unresolved. When comparing SRs of COPD patients with SRs of OHS patients in our study it becomes evident that one- and two-year survival of COPD patients is approximately the same as in OHS patients.

In our view, the positive prognostic effects of intensive therapy in COPD patients are associated with a compliance that can be rated as good, given a discontinuation rate of 18% within four years in COPD patients (compared to 21% in OHS patients), and an average daily use duration of 5.6h/d (5.2h/d in OHS patients) within the first year. However, this can be regarded as coincidental due to the low number of cases with analysable patient data after four years. In a large randomised study by Köhnlein *et al.* 2014 [5] using “High Intensity NIV” and providing 14 days of intensive contact with patients, a slightly higher average daily use duration of 5.9h/d and a lower discontinuation rate of 9.1% was achieved. Thus, we achieved similar compliance and a similar outcome using “High Intensity NIV”, however with significantly lower patient care expenditure. Our results on survival, depending on ventilation duration, indicate that consistent home ventilation, i.e. effective and long-term relief of the insufficient respiratory musculature, is prognostically relevant.

### Limitation of this Study

1. The effectiveness of ventilation therapy was documented by the extent of CO_2_ reduction under spontaneous breathing at the end of the inpatient stay at which ventilation parameters were set. CO_2_ follow-up examinations were carried out to verify ventilation effectiveness, but were not included in this study. Since the treatment protocol made provisions for intervention, under any circumstances, in case of progressive hypercapnia during the course of treatment, in our view, the influence of different CO_2_ concentrations over the course of treatment was considered irrelevant to the questions presented. Device-usage data were recorded only during the first year.
2. Obstructive sleep apnoea is common in patients with obese COPD. Most of COPD patients in this study were obese (mean BMI was 34.6). Some of them may have obstructive sleep apnoea. We know that approximately 90% of patients with OHS also have obstructive sleep apnoea. There may was a certain bias because of the influence of treated sleep apnoea on adherence or survival.
3. Adherence was recorded only during the first year of home ventilation. Clinical experience with our patient cohort regarding therapy acceptance has shown that compliance changes within the first 6 – 9 months. After that only minor changes in compliance were observed.

## CONCLUSION

In summary, our data show that “High Intensity NIV” in chronically hypercapnic COPD patients was efficient in routine care (with respect to CO_2_ reduction), and led to prognostically relevant survival rates that are similar to those reported earlier in randomised studies.

Furthermore, it was demonstrated that high intensity NIV in stable COPD leads to an effective relief of the respiratory musculature as measured by CO_2_ concentration is feasible and efficient even outside controlled clinical trials.

Under the “High Intensity NIV” regimen that we describe here, a significant, and in comparison with OHS patients, equally strong reduction of pCO_2_ values was achieved in COPD patients. Our data showed that with the selected ventilation procedure it is possible to achieve a CO_2_ reduction in COPD patients that is similar to OHS patients.

## Figures and Tables

**Table 1:** In- and exclusion criteria.

Inclusion Criteria	Exclusion Criteria
COPD	
Daytime pCO_2_ > 7,0kPa, ph > 7,35 FEV1/FVC < 80% Established diagnosis of COPD according to GOLD Stable on inhaled medication	Worsened dyspnoea, cough, increased sputum production two weeks before entry oft the study Clinical signs of an exacerbation [22] Any other cause for hypercapnia Decompensated cor pulmonale Systemic corticosteroids
Obesity Hypoventilation	
Daytime pCO_2_ > 7,0kPa, ph > 7,35 FVC < 70% BMI > 30kg/m^2^	FEV1/FVC < 80% Any other cause for hypercapnia Decompensated cor pulmonale

**Table 2:** Patient characteristics.

	COPD (n=51)		OHS (n=34)	
	mean	SE	mean	SE
Age at enrolment	66.9	1.3	65.4	1.8
Proportion of males (n; %)	32	63%	17	50%
BMI in kg/m^2^	34.6	1.4	45.4	1.4
6 MWT in m	206.8	17.9	190.8	24.8
FEV1 in l	1.1	0.1	1.4	0.1
FEV1 in %	42.7	2.5	54.8	3.0
rel. FEV1	0.6	0.0	0.8	0.0
VC in l	2.0	0.1	1.8	0.1
VC in %	55.1	2.1	55.2	2.9
RV in l	4.3	0.3	2.5	0.2
RV in %	191.2	12.7	114.9	7.6
ITGV in l	4.9	0.3	2.9	0.2
ITGV in %	155.5	8.4	97.2	6.9

6 MWT: 6-minute walk test distance, BMI: Body Mass Index, lung function parameters: please refer to abbreviations list. Shown are arithmetic means and standard errors (SE), unless indicated otherwise.

**Table 3:** Ventilation parameters in COPD and OHS.

	COPD n=51		OHS n=34		P Values
Number of patients with APCV, n (%)	49	96.1%	28	82.3%	0,340
Pinsp in cm H_2_O	22	3.7	22	3.9	0,43
PEEP in cm H_2_O	2.3	2.5	5.3	2.7	0,18
Number of patients with PEEP = 0, n (%)	21	41%	0	0%	0,026
Effective pressure in cm H_2_O	18.5	0.74	16.9	0.88	0,095
Ventilation rate in breaths/min	15.8	3.3	15.3	2.9	0,48
Prescribed duration of ventilation in h/d	11.8	2.4	11.1	1.9	0,51
Number of patients rejecting therapy, n (%)*	9	18%	7	21%	0,071
Average device usage in h/d *	5.6	4.4	5.2	3.2	0,17
Number of patients with pCO_2_ < 5.7kPa with NIV, n (%)	36	70.6%	21	61.8%	0,11

Shown are arithmetic means and standard errors, unless indicated otherwise.

Data were collected at discharge after initiation of NIV unless different stated.

Ventilation parameters are set parameters

* data achieved after 12 month NIV

Average device usage: calculated mean of daily increase of use-hours.

p-Values: non parametric test (Mann & Whitney) for independent samples.

**Table 4:** Ventilation effectiveness according to blood gas analysis parameters in COPD and OHS.

	Before NIV		With NIV		Diff.	95% CI of the difference		
	mean	SE	mean	SE	mean	lower	upper	p
COPD								
pH	7.36	0.06	7.45	0.04	-0.09	-0.11	-0.07	0
pCO_2_	8.7	1.5	5.8	0.8	2.83	2.34	3.29	0
pO_2_	8.0	1.8	10.2	1.4	-2.25	-2.94	-1.56	0
HCO_3_	32.4	6.0	30.2	3.7	2.24	0.67	3.80	0.006
BE	8.6	5.2	5.8	3.0	2.78	1.30	4.26	0
SO_2_	87.5	8.2	95.7	2.6	-8.2	-10.64	-5.75	0
6-MWT	206.8	110.4	267.0	108.4	60.2	81.7	38.8	0
OHS								
pH	7.37	0.06	7.45	0.05	-0.08	-0.10	-0.06	0
pCO_2_	8.5	1.2	5.9	0.8	2.55	2.12	2.98	0
pO_2_	7.6	1.3	10.0	1.7	-2.37	-2.99	-1.75	0
HCO_3_	32.7	4.9	30.0	4.0	2.65	0.98	4.32	0.003
BE	8.5	4.1	5.1	2.7	3.43	2.02	4.84	0
SO_2_	86.7	5.4	95.1	2.6	-8.34	-10.40	-6.35	0
6-MWT	190.8	124.0	259.0	137.4	65	93.7	36.2	0

Blood gas analysis at rest, spontaneous breathing.

Before NIV: Before initiation of ventilation; with NIV: before discharge with ventilation.

Values as mean, standard deviation, and difference: before NIV – with NIV; 6-MWT walk distance: with NIV – before NIV.

p: significance level, two-sided, (t-test for dependent samples).

**Table 5:** Survival of COPD and OHS patients: Univariate Analysis - Kaplan-Meier.

	COPD (n=51)		OHS (n=34)	
	%	SE in %	%	SE in %
1 year survival rate	83.3	5.4	85.1	6.2
2 year survival rate	72.8	6.8	72.1	8.0
3 year survival rate	55.2	8.1	68.3	8.4
Median survival time	47.3 Mo.	8.3 Mo.	Not reached	

## LIST OF ABBREVIATIONS

COPD: = chronic obstructive pulmonary disease
OHS: = obesity hypoventilation syndrome
NIV: = non invasive ventilation
CO_2_: = carbon dioxide
O_2_: = oxigene
FEV1: = forced expiratory volume in one / 1 second
VC: = vital capacity
FVC: = forced vital capacity
ABG: = arterial blood gasses
APCV: = assisted pressure controlled ventilation
pinsp: = inspiratory pressure
PEEP: = positive endexspirarory pressure
h: = hours
d: = day
pCO_2_: = carbondioxide pressure
pO_2_: = oxygene pressure
BMI: = body mass index
BE: = base excess
HCO_3_: = hydrogene bicarbonate
6 MWT: = six minute walking test
RV: = residual volume
ITGV: = intrathoracic gass volume
SO_2_: = oxygene saturation
SE: = standard error
CI: = confidence intervall
SR: = survival rate
